# PMM2‐CDG caused by uniparental disomy: Case report and literature review

**DOI:** 10.1002/jmd2.12122

**Published:** 2020-04-28

**Authors:** Laurien Vaes, George E. Tiller, Belén Pérez, Suzanne W. Boyer, Susan A. Berry, Kyriakie Sarafoglou, Eva Morava

**Affiliations:** ^1^ Faculty of Medicine KU Leuven Leuven Belgium; ^2^ Department of Genetics Kaiser Permanente Los Angeles California USA; ^3^ Center of Molecular Biology‐Severo Ochoa University Autonomous of Madrid, La Paz Institute for Health Research, Center for Biomedical Research on Rare Diseases Madrid Spain; ^4^ Department of Clinical Genomics Mayo Clinic Rochester Minnesota USA; ^5^ Division of Genetics and Metabolism, Department of Pediatrics University of Minnesota Medical School Minneapolis Minnesota USA; ^6^ Department of Pediatrics University of Minnesota Masonic Children's Hospital Minneapolis Minnesota USA; ^7^ Department of Clinical Genomics, and Department of Laboratory Medicine and Pathology Mayo Clinic Rochester Minnesota USA

**Keywords:** CDG, chromosome 16, congenital disorders of glycosylation, homozygosity, PMM2‐CDG, uniparental isodisomy, whole exome sequencing

## Abstract

**Background:**

Phosphomannomutase 2 deficiency **(**PMM2‐CDG) affects glycosylation pathways such as the N‐glycosylation pathway, resulting in loss of function of multiple proteins. This disorder causes multisystem involvement with a high variability among patients. PMM2‐CDG is an autosomal recessive disorder, which can be caused by inheriting two pathogenic variants, de novo mutations or uniparental disomy.

**Case Presentation:**

Our patient presented with multisystem symptoms at an early age including developmental delay, ataxia, and seizures. No diagnosis was obtained till the age of 31 years, when genetic testing was reinitiated. The patient was diagnosed with a complete maternal mixed hetero/isodisomy of chromosome 16, with a homozygous pathogenic PMM2 variant (p.Phe119Leu) causing PMM2‐CDG.

A literature review revealed eight cases of uniparental disomy as an underlying cause of CDG, four of which are PMM2‐CDG.

**Conclusion:**

Since the incidence of homozygosity for PMM2 variants is rare, we suggest further investigations for every homozygous PMM2‐CDG patient where the segregation does not fit. These investigations include testing for UPD or a deletion in one of the two alleles, as this will have an impact on recurrence risk in genetic counseling.

**SYNOPSIS**

Since homozygosity for pathogenic variants in PMM2 is rare, we suggest testing for uniparental disomy in patients with homozygous PMM2 variants, as this will have an impact on recurrence risk and genetic counseling.

## 1 INTRODUCTION

Congenital disorders of glycosylation (CDG) are a heterogenous group of metabolic diseases affecting the glycosylation process. Glycosylation plays a role in the post‐translational modification of most proteins in order to obtain proper intracellular localization and full functionality.^1^ The most common CDG is Phosphomannomutase‐2‐CDG (PMM2‐CDG), also previously known as congenital disorder of glycosylation type Ia. In this CDG pathogenic variants in the PMM2 gene lead to an impaired activity of phosphomannomutase 2,^2^, ^3^ an enzyme necessary for the biosynthesis of N‐glycoproteins.^4^, ^5^ Common findings in PMM2‐CDG patients include distinctive facial features, strabismus, hypoplastic cerebellum, ataxia, developmental delay, failure to thrive, coagulation abnormalities, and so forth.

Most CDG are inherited in an autosomal recessive manner.^6^ A minority are X‐linked or autosomal dominant. With the inclusion of testing parents' DNA into the accepted process of genetic variant analysis, de novo pathogenic variants causing CDG have also been identified.^7^ ALG13 was the first reported autosomal recessive inherited CDG due to de novo pathogenic variants.^7^ A de novo variant has only been described in combination with a pathogenic variant from one of the parents on the other allele.^8^ A de novo pathogenic variant is commonly found in dominant and X‐linked CDG^6^ as seen in SLC35A2‐CDG^9^, ^10^, ^11^, ^12^ or SSR4‐CDG.^13^

Typically, most PMM2‐CDG patients are compound heterozygotes. A few variants such as p.F119L p.D65Y, p.Y64C or p.T237M^3^, ^14^, ^15^ have been identified in the homozygous state. The fact that homozygous state is rare in PMM2‐CDG reflects on two fundamental aspects of PMM2‐CDG: it is a rare disease and the most common pathogenic R114H is thought to be incompatible with life.^16^ The variable geographic genotype distribution makes it difficult to determine a global homozygous genotype prevalence.^3^ International guidelines published in 2019 describe the homozygous genotype prevalence of some specific pathogenic variants such as a prevalence of 0.8% for the homozygous p.F119L variant.^17^ Homozygous variant prevalence described in population‐based studies ranged from 2.1% to 6.1%,^5^, ^17^, ^18^, ^19^, ^20^, ^21^ which indicates that homozygosity for PMM2‐CDG is rare.

We here report a homozygous PMM2‐CDG patient with maternal uniparental disomy (UDPmat) as the underlying cause.

## 2 CASE REPORT

Our patient was born at term to healthy nonconsanguineous parents after an uncomplicated pregnancy and vaginal delivery. Birth weight was 3203 g (36th percentile) and length 50.8 cm (72th percentile). Newborn screening results were normal. As an infant, developmental delay was noted by her mother, but no specific diagnosis was made after clinical evaluation and a karyotype at the age of 6 months showed normal results. The patient's speech was also noted to be significantly delayed. At an age of 20 months, she developed seizures associated with fever, which resolved by 4 years of age. She also presented at an early age with hearing loss, ataxia, microcephaly, retinitis pigmentosa, myopia, strabismus and short stature. She had several infections around the age of five.

At 6 years of age, she was diagnosed with cerebellar hypoplasia and short stature caused by panhypopituitarism treated with thyroid hormone, hydrocortisone and growth hormone supplements. When she reached the age of 10, she was again seen by a genetic consultant, who suspected autosomal recessive cerebellar parenchymal disorder type III based on her clinical symptoms. However, confirmatory molecular testing was not available at that time. At 21 years, osteoporosis of L1‐L4 was diagnosed.

## 3 GENETIC TESTING

At the age of 31, molecular testing was reinitiated. She had a normal 46,XX karyotype, comparative genomic hybridization and a capture panel for 30 genes associated with pontocerebellar hypoplasia. Single nucleotide polymorphism analysis was suspicious for uniparental disomy of chromosome 16 (UPD16) based on the discovery of two large Copy‐neutral Absence of Heterozygosity (CN‐AOH) regions.

Next, short tandem repeat analysis (STR) and whole exome sequencing were pursued to confirm UPD16 and to test our suspicion for an underlying genetic syndrome. Complete maternal mixed hetero/isodisomy of chromosome 16 was confirmed. A pathogenic variant of the PMM2 gene inherited from the patient's mother and also mapped on chromosome 16 (16p13) was found.^3^ Our patient was homozygous for the F119L (c.357C>A) due to UPD(16)mat and had thus PMM2‐CDG. After molecular testing, isoelectric focusing of serum transferrin demonstrated a type 1 pattern which was consistent with the molecular diagnosis of PMM2‐CDG.^18^

## 4 DISCUSSION

UPD is a genetic error occurring during gametogenesis and fertilization, whereby both copies of a chromosome are inherited from one parent.^19^, ^21^ UPD arises from nondisjunction of a chromosome pair in the germ cell of a parent during meiosis followed by a rescue event. There are two primarily common rescue events: trisomy rescue and monosomy rescue. Since most nondisjunction events take place during the first maternal meiosis, trisomy rescue will lead to UPDmat, and monosomy rescue will lead to paternal UPD.^20^ UPD is important to detect because it can unmask a recessive disease or lead to mosaicism and aberrant parental imprinting.^22^

Multiple uniparental disomies are associated with phenotypic abnormalities due to parent‐specific imprinting.^20^ An example of parental‐specific imprinting associated with UPD is maternal disomy of chromosome 7, seen in 10% of patients with Silver‐Russell syndrome.^20^ The clinical relevance of UPD(16)mat remains unclear as it has been found in healthy individuals as well as in patients with clinical conditions. The intrauterine growth retardation, cardiac, vascular, and skeletal malformations as well as facial dysmorphism frequently reported in UPD(16)mat patients may be due to low‐level trisomy 16 mosaicism.^23^ UPD(16)mat has also been reported in two patients with the Silver‐Russell phenotype.^24^

Seven imprinted genes located on chromosome 16 have been identified, although their function remains uncertain: SOX8, ZNF597, NAA60, SALL1, C16orf57, ACD, and FOXF1.^24^ There is still conflicting data regarding the imprinting of the FOXF1 gene(J.). Casanova et al suggested that FOXF1 is a highly dosage‐sensitive gene rather than a maternally imprinted gene^25^ whereas Schulze et al suggested that the phenotype difference between maternal and paternal UPD16 can be explained by the maternally imprinted FOXF1 gene.^26^ In contrast to UPD16mat, paternal UPD16 seems to have no clinical consequences.^27^

Another consequence of uniparental disomy is that it can cause recessively inherited disorders as in our patient.^22^ A literature review revealed that UPD has only been reported in five different CDG involving eight patients (see Table 1) of which only two patients (patients 1 and 5) are extensively described.^28^, ^32^ Schollen et al was the first to describe UPD in an ALG3‐CDG patient, whereby a de novo pathogenic variant in combination with segmental maternal isodisomy for chromosomal region 3q21.3‐3qter was found.^28^

**TABLE 1:** Overview of CDG patients reported with uniparental disomy, including our patient (patient 8)

	Patient 1	Patient 2	Patient 3	Patient 4	Patient 5	Patient 6	Patient 7	Patient 8
Type of CDG	ALG3‐CDG	PMM2‐CDG	PMM2‐CDG	B4GALT1‐CDG	POMT2‐CDG	PMM2‐CDG	GFPT1‐CDG	PMM2‐CDG
Homozygous mutation	p.R266C (c.796C>T): de novo	p.P113L (c.338C>T)	p.E139K (c.415G>A)	p.Y193X (c.579C>G)	p.E501A (c.1502A>C)	p.V231M (c.691AG>A)	c.865‐866insG Frame shift mutation	p.F119L (c.357C>A)
UPD	Maternal segmental uniparental isodisomy for chromosome 3 (q21.3‐qter)	Maternal isodisomy of 16 Mb and heterodisomy for the rest of chromosome 16	Maternal uniparental isodisomy for chromosome 16 (14.5 Mb region)	Maternal isodisomy for chromosome 9	Maternal segmental uniparental isodisomy for chromosome 14 (15 Mb region)	Paternal uniparental isodisomy for chromosome 16	Maternal segmental uniparental disomy for chromosome 2	Maternal mixed hetero/isodisomy for chromosome 16
Gender	Female	Female	Male	Female	Female	Female	Female	Female
Prenatal history	Negative	Not available	Cardiomyopathy detected at 38 weeks of gestation	Increased nuchal translucency and preeclampsia	Maternal tobacco use in first trimester	pregnancy complicated by inflammatory bowel disease		Negative
Family history	Negative			Negative	Negative	Negative		Negative
Duration of gestation	37 weeks			34 weeks	38 weeks	38 weeks	Full term	Full term
Neonatal Complication	Negative			Maternal hypertension and caesarian section	Negative	Bradycardia and poor feeding	Prolonged intubation	Negative
Clinical features	Microcephaly, dysmorphic features, clubfoot, epilepsy, truncal hypotonia, scoliosis, psychomotor developmental delay, optic atrophy	Ataxia, hypotonia, mild psychomotor retardation, severe cerebellar atrophy, coagulopathy, bursitis, strabismus, cutaneous hyper elasticity, pericardial effusion	Truncus arteriosus type II, developmental delay, hypotonia, strabismus, seizures, inverted nipples, lumbosacral fat pads, speech delay, gastroesophageal reflux, severe cerebellar atrophy and ventriculomegaly	Relative macrocephaly, hypertelorism, facial dysmorphism, respiratory distress, hypotonia, hepatosplenomegaly, failure to thrive, coagulopathy, hyperreflexia, inverted nipples, scalp and truncal angiomas and hypothyroidism	Muscle atrophy, generalized hypotonia, proximal muscle weakness, hyporeflexia, toe‐walking, waddling gait, Gowers maneuver, contractures, borderline low left ventricular ejection fraction and mild restrictive lung disease	Hepatomegaly, hypotonia, failure to thrive, microcephaly, inverted nipples, vulvar fat pads, cerebral and cerebellar hypoplasia, hepatic steatosis	Hypotonia, weakness with reduced spontaneous movement, areflexia	Developmental delay, speech delay, seizures, hearing loss, ataxia, microcephaly, retinitis pigmentosa, myopia, strabismus, short stature, cerebellar hypoplasia, panhypopituitarism, and osteoporosis of L1‐L4
Reference	Schollen et al^28^	Perez et al,^29^ and Perez‐Cerda et al^30^)	Perez‐Cerda et al^30^	Medrano et al^31^	Brun et al^32^	Tiller et al^33^	Krate et al^34^	

The molecular information of the remaining patients described in Table 1 was obtained through personal contact with the authors. Patient 2, besides being homozygous for PMM2 pathogenic variant (p.P113L(c.338C>T) due to UPD(16)mat had a de novo loss‐of‐function variant in the *COL5A1* gene, which is known to cause the Ehlers‐Danlos syndrome. Of note, patient 2 was the product of in vitro fertilization. Patient 3 was found to be mosaic through SNP arrays, with a wild type allele frequency (G) of 22% and a mutation allele frequency (A) of 77%. In patient 6, five anonymous markers from 16p13.3‐16q24.3 were consistent with paternal isodisomy (data not shown).

Patients 2 and 3 (Table 1) had overlapping phenotypic features also found in UPD(16)mat patients. Both presented with dermatologic involvement not typically seen in the clinical spectrum of PMM2‐CDG. The skin hyperelasticity in patient 2 relates to the COL5A1 mutation found in this patient.^35^ Cardiac problems have been previously described in PMM2‐CDG patients; therefore, it is unlikely that it is linked with UPD16.^36^ Bursitis was found in patient 2, which is also linked to the COL5A1 mutation. Patient 4 (B4GALT1‐CDG), patient 5 (POMT2‐CDG) and patient 7 (GFPT1‐CDG) had UPDmat of chromosome 9, 14 and 2, respectively, and all had a clinical phenotype consistent with CDG. Finally, looking at the diagnostic criteria for the Silver‐Russell syndrome, which was previously found in UPD(16) patients, patients 2, 3 and 8 with PMM2‐CDG due to UPD(16)mat did not fulfill the diagnostic criteria for Silver‐Russell syndrome, which has been associated with UPD(16)mat.

Since four out of the eight patients (patients 2, 3, 6, and 8) were found to have either a complete or segmental UPD(16)mat causing PMM2‐CDG, an argument could be made to look for UPD in homozygous PMM2‐CDG patients, as chromosome 16 is one of the most common chromosomes reported with UPD.^37^ Trisomy 16 is the most frequent prenatally detected trisomy and is embryonically lethal, unless trisomy correction takes place, giving rise to UPD16 mat.^38^

Another argument underscoring the importance of UPD studies when they are homozygous for a pathogenic variant, is that the occurrence of homozygous mutations in PMM2‐CDG is rare.^3^, ^16^ Combining the six population studies described in the introduction, an average prevalence of only 3.7 ± 1.52% was found for homozygosity in PMM2‐CDG patients.^5^, ^17^, ^18^, ^19^, ^20^, ^21^

## 5 CONCLUSION

The low prevalence of homozygosity among PMM2‐CDG patients together with the relative frequency of UPD16 suggests that uniparental disomy may be a plausible cause for many cases with homozygous PMM2 mutations. PMM2 variants require further evaluation when segregation analysis does not fit, in order to provide the most accurate genetic counseling for the family. An underlying deletion or uniparental disomy can be revealed in this way.

## CONFLICT OF INTEREST

The authors have declared that no competing interests exist.

## ETHICS APPROVAL

“IRB19‐005187, Clinical and Basic Investigations into Congenital Disorders of Glycosylation.”
